# Blood donor biobank as a resource in personalised biomedical genetic research

**DOI:** 10.1038/s41431-023-01528-0

**Published:** 2024-01-12

**Authors:** Jonna Clancy, Jarmo Ritari, Eevaleena Vaittinen, Mikko Arvas, Silja Tammi, FinnGen, Mikko Arvas, Jukka Partanen, Satu Koskela, Jukka Partanen

**Affiliations:** 1 Blood Service Biobank, Finnish Red Cross Blood Service, Vantaa, Finland; 2 Research and Development, Finnish Red Cross Blood Service, Helsinki, Finland

**Keywords:** Genetics, Medical research

## Abstract

Health questionnaires and donation criteria result in accumulation of highly selected individuals in a blood donor population. To understand better the usefulness of a blood donor-based biobank in personalised disease-associated genetic studies, and for possible personalised blood donation policies, we evaluated the occurrence and distributions of common and rare disease-associated genetic variants in Finnish Blood Service Biobank. We analysed among 31,880 blood donors the occurrence and geographical distribution of (i) 53 rare Finnish-enriched disease-associated variants, (ii) mutations assumed to influence blood donation: four Bernard-Soulier syndrome and two hemochromatosis mutations, (iii) type I diabetes risk genotype HLA-DQ2/DQ8. In addition, we analysed the level of consanguinity in Blood Service Biobank. 80.3% of blood donors carried at least one (range 0–9 per donor) of the rare variants, many in homozygous form, as well. Donors carrying multiple rare variants were enriched in Eastern Finland. Haemochromatosis mutation HFE C282Y homozygosity was 43.8% higher than expected, whereas mutations leading to Bernard-Soulier thrombocytopenia were rare. The frequency of HLA-DQ2/DQ8 genotype was slightly lower than expected. First-degree consanguinity was higher in Blood Service Biobank than in the general population. We demonstrate that despite donor selection, the Blood Service Biobank is a valuable resource for personalised medical research and for genotype-selected samples from unaffected individuals. The geographical genetic substructure of Finland enables efficient recruitment of donors carrying rare variants. Furthermore, we show that blood donor biobank material can be utilised for personalised blood donation policies.

## Introduction

Large biobank genome data collections combined with electronic health records have made phenome-wide association studies (PheWAS) feasible [1] leading to increased power and novel discoveries in disease genetics [2]. Blood donors have been suggested to be an excellent option for large cohorts of healthy individuals [3] as blood donors voluntary and frequently donate blood. Based on in-depth interviews blood donors are known to have a positive attitude towards scientific research and for use of their donated samples for research if not needed for patient care [4, 5]. The blood donor population, however, is highly selected due to prior health questionnaires and healthy donor effect [6] referring to accumulation in the blood donor pool of individuals with a very good health status. The healthy donor effect, an example of membership bias, may lead to severe confounding effects in research settings if not considered.

As a result of genetic drift, isolated populations, such as the Finnish population, encounter bottlenecks that may enrich both deleterious [7], disease-predisposing alleles and disease-protective alleles. Small founder population, geographical and linguistic isolation [8, 9] and a strong bottleneck approximately 120 years ago, are manifested in the Finnish disease heritage, a set of recessive diseases that are more common in Finland than elsewhere [10] and also to a remarkably lower prevalence of other diseases, such as cystic fibrosis. In addition, the significantly lower genetic diversity of the Finns compared to other European populations [11] enable more efficient genetic research compared to populations with more heterogeneous inheritance. It is worth noting, that even though homogeneous compared to other populations, a strong genetic variation occurs within Finland, leading to geographical genetic substructure of the population [12].

To evaluate the usefulness of blood donor-based biobank for genetic studies, we investigated the occurrence and geographical distribution of three set of genetic variants: (1) rare Finnish-enriched variants associated with susceptibility to many multifactorial diseases identified previously by FinnGen project [13] (2) blood donation-related variants: mutations causing hemochromatosis, an iron accumulation disease treated with phlebotomy, and Bernard-Soulier syndrome, a rare macro thrombocytopenia caused by defective proteins important to thrombocyte development and function, (3) HLA-DQ2/DQ8 heterozygotes, established to have a high susceptibility to type I diabetes, an autoimmune, multifactorial disease enriched in Finland. A few founder mutations for hemochromatosis are shared by all Northern European populations whereas a set of local mutations for Bernard-Soulier syndrome explain the majority of the Finnish cases [14, 15]. Individuals with hemochromatosis mutations can be assumed to be enriched to and those with Bernard-Soulier syndrome missing from blood donor pool. The frequency of type 1 diabetes risk haplotype, HLA-DQ2/DQ8 is assumed to be lower among blood donors than in the general population. Furthermore, we investigated the geographical distribution of the variants in Finland and the degree of consanguinity in Blood Service Biobank. In the present study we evaluate how well a blood donor-based biobank encompasses the rare and common genetic variants and certain HLA-haplotypes in Finland to understand the actual value of blood donor cohorts in studies of disease-associated variants and personalised genetics.

## Material and methods

### Samples, genome data and blood donation data

31,880 blood samples were collected along the standard blood donation from blood donors who had given a written biobank consent for the Blood Service Biobank of the Finnish Red Cross Blood Service, Finland. Use of the samples and data is in accordance with the biobank consent and meets the requirements of the Finnish Biobank Act 688/2012.

The biobank samples were genotyped as part of the FinnGen project with the FinnGen ThermoFisher Axiom custom array v1 or v2. Genotyping, quality control, and genome imputation protocols, R11, are described in detail in FinnGen Gitbook [16]. In brief, genotype calling was performed with AxiomGT1 algorithm. Prior the imputation, genotyped samples were pre-phased with Eagle 2.3.5 with the default parameters, except the number of conditioning haplotypes was set to 20,000. Genotype imputation was performed using the population-specific imputation reference panel SISu v3 including 3775 high coverage (25–30x) whole genome sequence data, with Beagle 4.1 (version 08Jun17.d8b). Genotypes were then returned to Blood Service Biobank.

The principal component analysis, PCA, was performed as part of FinnGen project for the analysis of population structure and is described in detail in FinnGen Gitbook [16]. After variant filtering and LD pruning, the FinnGen data was merged with 1k genome project (1kgp) data to filter out outliers. The final values of 20 principal components were then returned to Blood Service Biobank.

Blood donation data contains the blood donation-related information, birth year, ABO blood group, haemoglobin value, home region and donation history. This information was used in the analyses when applicable.

Comparison between Blood Service Biobank (*N* = 35,709) and FinnGen (*N* = 292,432) cohorts for allele frequencies of the rare variants, level of kinship and mean values of principal components 1 and 2 were performed with R7 dataset [16].

### Variant identification

Genotypes were called from VCF files under the following conditions: when imputed dosage score was ≥0 or ≤0.1, dosage value was considered as 0, when imputed dosage score was ≥0.9 or ≤1.1, dosage value was considered as 1 and finally when imputed dosage score was ≥1.9 or ≤2.0, dosage value was considered as 2. Samples which met these conditions in case of all 58 variants were included in the study.

The occurrence and geographical distribution of the following variants were analysed: (i) 53 rare disease-associated variants [13]; (ii) two mutations in HFE gene, H63D and C282Y, two Finnish-enriched mutations in GP1BA gene (Leu129Pro, Tyr518Leufs∗83) and two in GP9 gene (Asn45Ser and Leu40Pro) leading to Bernard-Soulier thrombocytopenia [14, 15, 17] that can be assumed to influence blood donation activities; and (iii) HLA-DQ2/DQ8 heterozygotes, HLA-DRB1*03:01-DQA1*05:01-HLA-DQB1*02:01 and HLA-DRB1*04:01-DQA1*03:01-DQB1*03:02, who have a high risk for type I diabetes [18, 19]. These two HLA-haplotypes include the known predisposing risk factors for type 1 diabetes: amino acid change in HLA-DRB1 molecule at position 13 and 71 and in HLA-DQB1 molecule at position 57 ref. [18]. All the investigated variants included in the study are listed in Supplementary Table 1.

### Consanguinity

Relatedness between the donors had been previously determined in FinnGen project [16]. The level of consanguinity in each cohort, Blood Service Biobank and FinnGen, was calculated as following: 10,000 random samples were extracted in each cohort and unique study donors with kinship relationships within the cohorts were extracted. This was repeated 1000 times to increase the trustworthy of real-life situation. The percentage of individuals with first-, second- and third-degree consanguinity in each cohort were calculated.

### HLA imputation and haplotyping

HLA-A, -B, -C, -DRB1, -DQA1, -DQB1, -DPB1 alleles were imputed at high-resolution level using HIBAG algorithm [20] with population-specific models in genome build 38 as described by Ritari et al. [21]. HLA-haplotyping was performed on high-resolution level on imputed HLA-alleles with Gap R package version 1.2.1. The mean posterior probability, PP, value of the imputed HLA-alleles was calculated prior haplotyping and all the samples where the mean PP value of all imputed HLA-alleles was 0.80 or higher, were included in haplotyping. The cut-off PP value of HLA-haplotypes was set to 0.5. Four donors had more than one possible haplotype combination which were not available in the reference haplotype lists [22] (FRCBS unpublished). These donors were excluded from the HLA-haplotype analyses. Altogether HLA-haplotypes from 29,659 donors (59,318 haplotypes) were available for the analysis.

### Statistical testing

The allele frequencies of rare disease-associated variants between Blood Service Biobank and FinnGen cohort were compared by linear regression. Student’s *t*-test was used to compare the mean values of the principal component 1, PC1, and principal component 2, PC2, within the blood donor cohort and between the two cohorts. Linear regression was used to model the response of HFE C282Y genotype, age and lifetime donation count (donors with ≤ 3 donations were filtered out) to the donor’s mean haemoglobin level (measured at the point of each blood donation event). Hardy-Weinberg equilibrium, HWE, was calculated to all variants in the blood donor cohort by the Hardy-Weinberg R package (HWExact or HWChisq), version 1.7.5. Chi-squared test and Fisher’s exact test were used to analyse the significance of the consanguinity level differences between Blood Service Biobank and FinnGen. P-value adjustment was performed with Stats R package using method “BY”.

All analyses were performed in R [23] version 3.6.1 or later, with R Studio [24].

## Results

### Demographics of the Blood Service Biobank

We compared the demographics of the Blood Service Biobank cohort to 203,861 blood donors who had donated blood in Finland during the same period as the biobank cohort was collected (Table 1). Overall, we could see several differences between the two cohorts.

**Table 1:** Demographics of the Blood Service Biobank cohort and non-Biobank Blood Donor pool (starting 1.11.2017).

	Blood Service Biobank cohort *N* = 31,880		non-Biobank blood donor pool starting 1.11.2017 *N* = 203 861		X² comparison between the two cohorts	
	Female	Male	Female	Male	Female	Male
	% (*N*)	% (*N*)	% (*N*)	% (*N*)	*p-value*	*p-value*
Sex	59.5 (18,961)	40.5 (12,919)	59.8 (121,818)	40.2 (82,043)	1.00	1.00
Age						
1946–1955	8.7 (1642)	13.1 (1692)	4.5 (5495)	6.2 (5087)	1.96E–127	8.72E–174
1956–1965	20.0 (3799)	26.2 (3389)	13.2 (16,056)	15.2 (12,462)	2.89E–138	1.89E–212
1966–1975	18.5 (3506)	20.7 (2677)	15.6 (19,014)	17.2 (14,074)	3.40E–22	2.14E–21
1976–1985	17.2 (3261)	16.9 (2187)	17.7 (21,607)	18.0 (14,796)	7.21E–01	2.96E–02
1986–1995	25.1 (4760)	18.5 (2391)	25.7 (31,289)	23.4 (19,201)	8.65E–01	3.58E–33
1996–2005	10.5 (1993)	4.5 (583)	23.3 (28,355)	20.0 (16,420)	0.00E+00	0.00E+00
Lifetime donation activity						
Less than 5	13.5 (2563)	7.8 (1004)	53.2 (64,762)	50.5 (41,400)	0.00E+00	0.00E+00
5–14	35.6 (6756)	22.7 (2932)	26.2 (31,936)	22.2 (18,199)	1.61E–158	1.00E+00
15–29	24.8 (4694)	22.1 (2856)	12.1 (14,737)	12.5 (10,264)	0.00E+00	2.30E–187
30 or more	26.0 (4948)	47.4 (6127)	8.5 (10,383)	14.8 (12,180)	0.00E+00	0.00E+00
ABO						
A+	33.2 (6302)	34.5 (4462)	32.4 (39,416)	33.3 (27,330)	1.87E–01	7.31E–02
A-	6.5 (1239)	6.4 (830)	5.5 (6728)	5.2 (4298)	4.41E–07	6.43E–07
AB+	5.6 (1058)	5.7 (730)	6.0 (7321)	6.2 (5078)	2.29E–01	2.06E–01
AB-	1.2 (236)	1.1 (148)	1.1 (1341)	1.1 (877)	8.48E–01	1.00E+00
B+	13.4 (2550)	12.9 (1676)	13.6 (16,515)	13.7 (11,199)	1.00E+00	3.98E–01
B-	2.9 (544)	2.9 (372)	2.4 (2864)	2.3 (1899)	2.86E–04	1.52E–03
O+	29.2 (5544)	28.8 (3717)	27.8 (33,925)	27.9 (22,888)	1.13E–03	4.23E–01
O-	7.6 (1444)	7.5 (973)	6.1 (7402)	5.6 (4635)	1.53E–14	1.36E–15
NA	0.2 (44)	0.1 (11)	5.2 (6306)	4.7 (3839)		
Donor’s home region (self reported)						
Kainuu	0.4 (68)	0.4 (50)	1.1 (1350)	1.2 (981)	3.65E–20	7.46E–15
Lapland	0.8 (159)	0.8 (100)	1.9 (2299)	1.7 (1414)	6.38E–23	4.68E–14
Ahvenanmaa	0.9 (178)	0.8 (97)	0.8 (914)	0.7 (594)	7.91E–02	1.00E+00
Central Ostrobothnia	0.8 (143)	1.2 (161)	1.1 (1337)	1.3 (1082)	3.07E–04	1.00E+00
North Karelia	1.3 (238)	1.3 (171)	2.1 (2505)	1.8 (1503)	3.93E–12	7.80E–04
Ostrobothnia	1.6 (300)	2.0 (259)	3.0 (3666)	3.7 (3012)	1.40E–26	2.26E–20
Southern Savo	1.7 (327)	2.0 (260)	1.6 (1959)	1.5 (1253)	1.00E+00	7.49E–04
Kymenlaakso	1.9 (361)	1.8 (231)	2.4 (2982)	2.4 (2005)	9.27E–05	9.65E–05
South Karelia	2.1 (402)	1.7 (214)	2.0 (2411)	1.9 (1521)	1.00E+00	1.00E+00
Kanta-Häme	2.3 (439)	2.5 (324)	3.1 (3725)	3.4 (2760)	4.41E–07	7.07E–06
Satakunta	3.5 (654)	3.8 (485)	3.2 (3868)	3.3 (2740)	5.02E–01	1.90E–01
South Ostrobothnia	4.1 (771)	4.7 (609)	3.2 (3912)	3.6 (2943)	2.52E–08	9.03E–09
Northern Ostrobothnia	4.8 (908)	5.8 (751)	6.6 (8088)	7.3 (5985)	1.45E–20	2.56E–08
Keski-Suomi	5.9 (1125)	6.0 (770)	5.6 (6806)	5.3 (4319)	5.75E–01	1.51E–02
Northern Savo	6.1 (1165)	6.5 (840)	4.0 (4911)	3.7 (3055)	1.65E–38	1.58E–47
Päijät-Häme	5.8 (1104)	5.8 (745)	3.4 (4167)	3.4 (2826)	5.71E–57	4.02E–36
Pirkanmaa	10.9 (2074)	11.4 (1473)	11.5 (14,005)	11.3 (9263)	2.73E–01	1.00E+00
Varsinais-Suomi	17.9 (3393)	16.7 (2159)	9.3 (11,285)	9.0 (7388)	4.69E–284	3.86E–159
Uusimaa	26.8 (5083)	24.6 (3176)	33.2 (40,477)	32.3 (26,495)	4.34E–67	4.36E–67
Unknown	0.4 (69)	0.3 (44)	0.9 (1151)	1.1 (904)		

In both cohorts, the overall number of female donors was higher, but male donors had higher donation activity. The age and donation activity distributions differed between the two cohorts; especially high difference was seen in the donation activity of young men. Blood group distribution between the two cohorts showed modest differentiation in all the ABO Rh blood groups, apart from AB– in both sexes and B+ in women. Highest difference was seen in O– distribution. All the provinces in Finland were represented in the study cohort (Table 1).

**Fig. 1:**
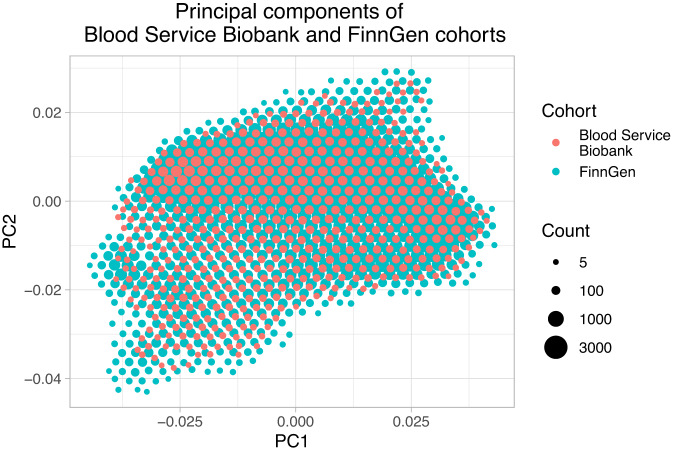
Principal components 1 and 2 of the Blood Service Biobank (*N* = 34,619) and FinnGen (*N* = 274,535) cohorts. Each point has been created by binning bivariate data points, PC1 and PC2, of the donors into a bin by R package Hexbin. Minimum count in each bin is 5.

The mean values of PC1 and PC2 were compared by *t*-test between the Blood Service Biobank and FinnGen cohort. There was a statistically significant albeit small difference between the mean values of the PC1s of the two cohorts (*p* = 6.89e–15). The difference explained only 2% of the SD of the whole study cohort. The PC1 component is assumed to reflect the east-west axis of the Finnish population [12]. No difference was seen in PC2 comparison (*p* = 1.00). Principal components 1 and 2 of the two cohorts are shown in Fig. 1.

### Genetic composition of the Blood Service Biobank

**Fig. 2:**
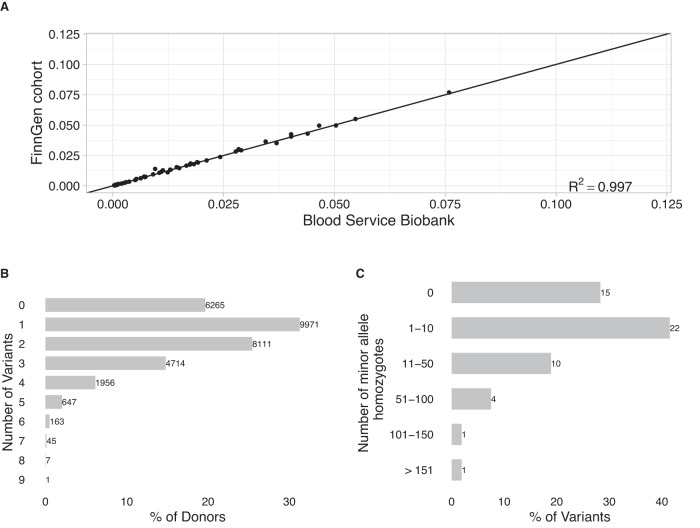
Fifty-three rare Finnish enriched disease-associated variants in Blood Service Biobank. **A** Linear regression equation of the minor allele frequencies in Blood Service Biobank and FinnGen cohort. **B** Amount of variants carried by donors in Blood Service Biobank **C** Amount of the 53 variants homozygous for the minor allele in blood donor Biobank.

Based on linear regression, we could see a strong relationship between the allele frequencies of the 53 disease-associated variants in Blood Service Biobank and FinnGen cohort (adjusted *R*² = 0.997, *p* = 6.39e–15, Fig. 2A). Hence the alleles were found in the Blood Service Biobank cohort with similar frequencies as in the FinnGen cohort, consisting mostly of hospital-based biobanks.

All the investigated rare variants were found among the blood donors. 80.3% (*N* = 25,615) of the blood donors in the Biobank had at least one of the 53 rare variants, the range was 0–9. One blood donor carried 9 different rare variants (Fig. 2A). No less than 23.6% (*N* = 7533) of them carried three or more variants. Of the 53 rare variants, 38 were found at least once as homozygous (Fig. 2C). For 16 variants more than 10 homozygotes were found in the Blood Service Biobank. More than hundred homozygotes were found in two variants, rs144651842 and rs77482050. rs144651842 has been shown to be protective against chronic lower respiratory diseases and rs77482050 protective against prostate cancer [13].

Two of the rare variants were not in the nominal (*p* < 0.05) Hardy-Weinberg Equilibrium: 17 minor allele homozygotes were found as compared to the 9 assumed for protective variant against arthrosis rs35937944, and 5 minor allele homozygotes found as compared to 2 assumed for rs201483470, a variant associated with coronary revascularization [13]. However, after adjustment the differences did not meet statistical significance.

As expected, the two hemochromatosis related mutations HFE H63D and HFE C282Y had higher frequencies in Blood Service Biobank than in the general population; H63D: MAF Blood Service Biobank 0.1164, MAF gnomAD (FIN) 0.09594, C282Y: MAF Blood Service Biobank 0.0390, MAF gnomAD (FIN) 0.0356 [25, 26]. There were 434 homozygotes (433 expected) for HFE H63D and 69 homozygotes (48 expected) for HFE C282Y. HFE C282Y homozygotes were found 43.8% more than expected. The observed number of donors found as compound heterozygotes (*N* = 284) for the two HFE mutations corresponded to the expected number (*N* = 290).

As expected, Bernard-Soulier Syndrome causing mutations were found only in heterozygous form in Blood Service Biobank. The minor allele frequencies of GP1BA Leu129Pro, MAF 0.0009, and GP9 Leu40Pro, MAF 0,0002, were lower in the Blood Service Biobank than the allele frequencies in the general population 0.0015 and 0.001 ref. [25, 26], respectively. The number of donors heterozygous for GP1BA Leu129Pro mutation, *N* = 55, and GP9 Leu40Pro mutation, *N* = 14, were close to the expected, *N* = 57 and *N* = 13, respectively. Bernard-Soulier mutation GP9 Asn45Ser was found in Blood Service Biobank with higher minor allele frequency, MAF 0.0008, than in the general population, MAF 0.00038 (ref. 25, 26). The number of donors heterozygous for this mutation, *N* = 49, was close to the expected, *N* = 51, in the Hardy-Weinberg Equilibrium. As the Bernard-Soulier mutations are rare and some of them are single-family mutations, it was not surprising that one mutation, Tyr518Leufs∗83, of the four was not included in the genotyping array.

Finally, we studied the occurrence of type I diabetes risk genotype HLA-DQ2/DQ8 in the Blood Service Biobank. HLA-DQ2/DQ8 heterozygous blood donors were observed 14.7% less than expected (expected *N* = 375, observed *N* = 320), however, the difference was not significant (*p* = 4.1e–01).

### Level of haemoglobin and HLA-haplotypes of the HFE C282Y homozygotes

Linear regression model showed male gender and HFE C282Y homozygosity to be associated with higher haemoglobin level (*p* < 2e–16, *p* = 3.33e–09, respectively), whereas age and lifetime blood donation count was shown to be negatively associated with haemoglobin level (*p* < 2e–16, *p* = 3.98e–12, respectively) when mean haemoglobin levels of HFE C282Y homozygous and wild type donors were compared (Supplementary Fig. 2). HFE C282Y homozygosity increased the haemoglobin level 5.6 g/L (95% CI 3.86–7.25). Model fit well to the dataset (F (4,28336) = 8035, *p* < 2.2e–16) and the variables, sex, HFE C282Y genotype, age and lifetime blood donation count, explained the haemoglobin level moderately, adjusted *R*^2^ = 0.53.

As HFE C282Y maps close to the HLA segment on chromosome 6 and as hemochromatosis is known to show in European populations associated with HLA A3, B7 ref. [27, 28], we also investigated the HLA-haplotypes of the HFE C282Y homozygotes to see whether the HLA associations are similar in Finland. Altogether 79 different HLA-A to HLA-DPB1 haplotypes were found among the donors homozygous for HFE C282Y. HLA-A*03:01 allele was seen in altogether in 53.8% of the HFE C282Y homozygotes and in homozygous form in 21.4%. HLA-A*03:01-B*07:02 haplotype was seen in 33.1% in these homozygotes and in homozygous form in 9.3%. The most common HLA-haplotype from A to DQB1 was A*03:01-B*07:02-C*07:02-DRB1*15:01-DQA1*01:02-DQB1*06:02, found as heterozygous in 19.2% of the HFE C282Y homozygotes, whereas 7.5% carried this haplotype in the Blood Service Biobank cohort.

### Geographical distribution of the markers in the Blood Service Biobank

When comparing the mean values of PC1 between those hetero- or homozygous for the minor alleles and those homozygous for the major allele, 46 variants showed statistically significant differences (*p* < 0.05). The corresponding result for PC2 was 31. The individual PC1-PC2 plots for each variant are shown in Supplementary Fig. 3. Supplementary 4 shows the mean PC1 and PC2 values in relation to geographical location in Finland based on blood donor’s region of residence.

**Fig. 3:**
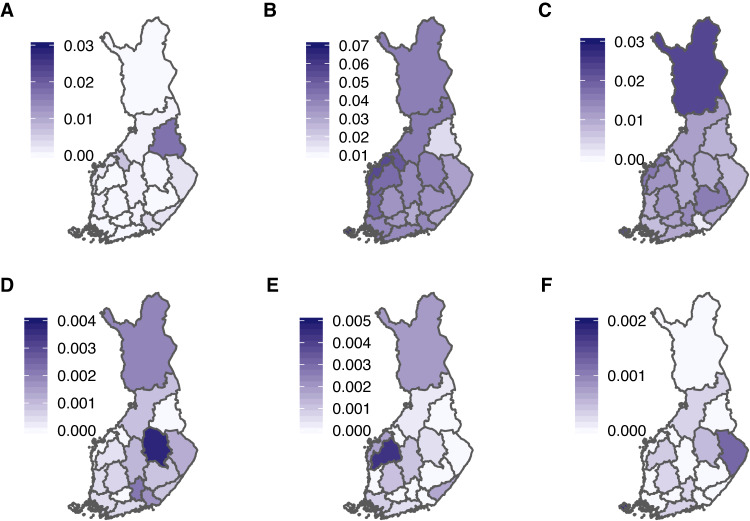
Distribution and geographical enrichment of blood donors in Blood Service Biobank. Distribution of blood donors carrying **A** multiple rare disease-associated genetic variants **B** HFE C282Y mutation **C** T1D risk haplotype HLA-DQ2/DQ8, **D** GP1BA Leu129Pro mutation, **E** GP9 Asn45Ser mutation, and **F** GP9 Leu40Pro mutation.

We could see geographical enrichment in the overall frequency of the 53 rare variant carriers, HFE C282Y, GP1BA Leu129Pro, GP9 Asn45Ser and GP9 Leu40Pro mutations as well as HLA-DQ2/DQ8 heterozygotes in Finland (Fig. 3). Donors carrying multiple rare variants were enriched in the Kainuu region of the Eastern Finland. The frequency of HFE C282Y mutation was relatively high in the whole country except in Kainuu region. In addition, we could see enrichment of this mutation in Ahvenanmaa and West of Finland. GP1BA LeuPro129 was enriched in the Northern Savo region of the Eastern Finland and GP9 Asn45Ser was enriched in South Ostrobothnia. GP9 Leu40Pro mutation was found only in 14 donors as heterozygous in Uusimaa, North Karelia, Pirkanmaa, Päijät-Häme, Northern Savo, South Ostrobothnia, Northern Ostrobothnia, and Ahvenanmaa regions. Bernard-Soulier syndrome causing mutations were not met in several regions at all. HLA-DQ2/DQ8 heterozygotes were enriched in Ahvenanmaa and Lapland. Frequencies of the mutations and HLA-DQ2/DQ8 heterozygotes in each region are listed in Supplementary Table 1.

### Consanguinity

**Fig. 4:**
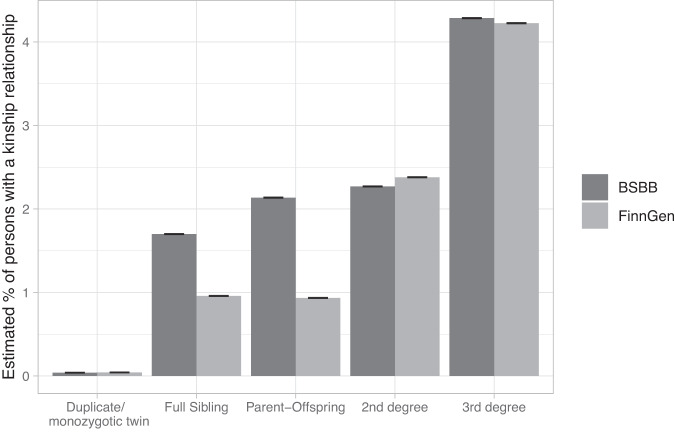
Consanguinity in Blood Service Biobank. The degree of consanguinity is higher in blood donor cohort than in FinnGen cohort for full siblings and parent-offspring. BSBB = Blood Service Biobank. Standard deviation of the mean value in each consanguinity group in both cohorts is shown.

The degree of consanguinity in the Blood Service Biobank and FinnGen cohort was compared for the first-, second- and third-degree relatives. The level of consanguinity was higher in the Blood Service Biobank in the first-degree relatives; 1.7% (SD 0.0017) were full siblings and 2.14% (SD 0.0019) had parent-offspring relationship whereas the corresponding figures in the FinnGen cohort were 0.96% (SD 0.0013) and 0.94% (0.0014), respectively (Fig. 4). Monozygotic twins/duplicates were met slightly more often in the FinnGen cohort, 0.042% (SD 0.0003) than in the Blood Service Biobank cohort 0.039% (SD 0.0003). Differences in the first-degree relatives were statistically significant in full sibling (*p* = 1.07e–04) and parent-offspring level (*p* = 1.91e–10) but not in case of the monozygotic twins/duplicates (*p* = 1.0). This could be the result of twin cohorts included in the FinnGen study [29]. Second degree relatives were seen slightly less in Blood Service Biobank cohort, 2.3% (SD 0.0020), than in the FinnGen cohort, 2.4% (SD 0.0022), while the third-degree relatives were met more often in the Blood Service Biobank, 4.3% (SD 0.0026), than in the FinnGen cohort, 4.2% (SD 0.0029). There was no statistically significant difference in second- or third-degree relativeness between the two cohorts, *p* = 1.0 and *p* = 1.0, respectively.

## Discussion

Blood donor biobank is considered to be a valuable resource in research due to the positive attitude towards research of blood donors as well as the voluntary and repeated blood donations. Samples from patients are an obvious target for many disease-focused studies and healthy blood donors carrying a desired gene variant can provide an excellent starting material for cellular and molecular biology studies without confounding effects of medication or disease progression. However, if not taken into consideration, the healthy donor effect may lead to membership bias and thus further to unpredictable consequences [6, 30].

We could see statistically significant differences between the Biobank and blood donor cohorts. There was a difference between age and donation activity distributions; this may reflect the high-level commitment of regular and usually older donors toward extra requests by the Blood Service, such as biobanking, and the fact that first-time donors are rarely recruited to the biobank. Hence, in the biobank cohort, the age distribution is focused on the older age which then also reflects on to the overall lifetime donation activity. Biological reasons may reflect to the lifetime donation activity of women. Lack of young men in Biobank cohort may be explained by recruiting practises; military service is mandatory for men in Finland, but donors are not recruited to Biobank in blood donations taking place in the defence forces. A high difference in O- blood group distribution could be a result of higher donation activity of biobank donors in general or because donors with O- blood group are relatively active and committed donors, hence they are presumably recruited to biobank more often. The number of samples from more remote and sparsely populated areas of Finland, such as Kainuu and Lapland, were low due to blood collection and biobank recruitment practices. In turn, the South-West area (Varsinais-Suomi) was over-represented in the Biobank as the result of active local recruitment.

The Finnish population is one of the most studied populations due to its unique genetic inheritance [7, 8, 29, 31]. Isolated populations that have undergone strong genetic bottlenecks like Finland, provide an opportunity to find and study genetic variants that have a strong effect in disease susceptibility and that are rare in most other populations [7, 29]. The impact of the Finnish settlement history can still be seen in the distribution of genetic variation in Finland today [12, 32, 33]. The Kainuu region has been utilised previously in several genetic studies due to the region’s unique genetic inheritance [34]. In the present study we show that the overall rare disease-associated variant frequency was highest in the Kainuu region, whereas the frequency of HFE C282Y mutation was lower in Kainuu region. Furthermore, the thrombocytopenia causing mutations GP1BA Leu129Pro, Asn45Ser and Leu40Pro weren’t met at all in Kainuu (Fig. 3). The occurrence of predisposing risk factor for type 1 diabetes, HLA-DQ2/DQ8 heterozygosity, was enriched in Ahvenanmaa and Lapland, whereas GP1BA Leu129Pro mutation was mainly seen in Northern Savo and GP9 Asn45Ser in South Ostrobothnia. These differences are largely due to the settlement history in Finland; early settlement, before A.D 1550, focused on the southern part of the country mainly by people from Scandinavia. Late settlement took place in 16th and 17th century when small family groups from the South migrated to the East and Northern parts of the country [8, 12, 35]. The genetic influence of these founder populations in East and West of Finland [12, 35] is reflected in the findings of the present study; genome level clustering either in East or West of Finland was seen in the carriers of several different genetic variants. In addition, the HFE C282Y mutation has been shown to originate in Europe [36] or possibly in Ireland or Scandinavia [37]. Taken this and the early settlement history of Finland into consideration, we could still see the route of this mutation in the present study; although present in all regions in Finland, HFE C282Y mutation was enriched in Ahvenanmaa and South Ostrobothnia regions and was met in lowest frequency in Kainuu region. Furthermore we show, that donors homozygous for this mutation carried a well-known hemochromatosis haplotype marker, HLA-A*03:01(refs. 27, 37), and that HLA-haplotype A*03:01-B*07:02-C*07:02-DRB1*15:01-DQA1*01:02-DQB1*06:02 was enriched in these donors. The latter is interesting in terms of special immunological features described in patients with clinical hemochromatosis [38].

HFE C282Y mutation as homozygous, the most common cause of hereditary hemochromatosis, causes organ damage level hemochromatosis only for a minority of its carriers, leaving room for other hepcidin regulator genes, such as TfR2 and HJV [39], and contributing factors, such as modifier genes and dietary factors [39, 40]. Identification of HFE C282Y homozygotes among blood donors may have some practical consequences for blood banks: homozygotes perhaps should avoid iron supplementation after blood donation [41] and determination of their ferritin and transferrin levels may be recommended [42]. Informing donors homozygous for HFE C282Y mutation is supported by the recommendation of American College of Medical Genetic and Genomics [43]. We are currently setting up a policy how to communicate these findings to blood donors.

Regarding the mutations causing Bernard-Soulier syndrome, GP1BA Leu129Pro, GP9 Asn45Ser and Leu40Pro no homozygous donors were found, as expected. There is no obvious reason for the higher minor allele frequency of GP9 Asn45Ser in Blood service Biobank. As this mutation is enriched in certain areas in Finland, understanding the geographical distribution of the reference dataset would be necessary. Individuals heterozygous for Bernard-Soulier Syndrome causing mutations, are measured usually half of the normal GP Ib-IX-V expression level but display only mild symptoms, such as mild bleeding [44]. It may be relevant to further investigate whether blood donation is a harmless event to these individuals or whether the blood products from individuals heterozygous for Bernard-Soulier Syndrome mutations differ from blood products of donors with no copy of these mutations.

In addition to altruism [45], having blood donors in the family has been shown to effect on blood donation motive [46]. We show, to our knowledge the first time, that the degree of first-degree consanguinity is higher among blood donors when compared to population of non-blood donors. This finding is supported by the earlier findings of “family tradition” as a motivation for blood donation [45, 46]. The greater degree of first-degree relatives enables the use of the material in linkage and trio-based analysis. In this study we wanted to understand the structure of the biobank cohort, hence the first-degree relatives were included in the study.

In the present study we focused on a limited number of variants associated with diseases, some of them being rare and enriched in Finland. More comprehensive and systematic studies considering all genetic factors potentially related to traits affecting blood donation eligibility, such as body weight, haemoglobin level or blood group systems, would be needed to fully understand the actual value, or possible bias, of blood donors in disease-associated genetic studies or as a control group in general. Also, it is worth keeping in mind that biobank donors are active and regular blood donors, hence we must be careful of making assumptions of them representing the general non-blood donor population.

The results of this study clearly indicate that for most of genetic variants and polymorphisms, even for those with a relatively strong effect size for diseases, the blood donor biobank provides a good and useful cohort for a genotype-based collection of samples. Despite of the selection of healthy individuals as blood donors, the blood donor biobank was found to include most of disease-associated gene variants and polymorphisms with frequencies equal or near to those found in the patient enriched FinnGen cohort.

## Supplementary information

Supplementary Table 1a

Supplementary Table 1b

Supplementary Table 1c

Supplementary Figure 2

Supplementary Figure 3

Supplementary 4a

Supplementary 4b

Supplementary 4c

Supplementary Table 5

Supplementary Table and figure legend

## Data Availability

The data that support the findings of this study are available from the Finnish Red Cross Blood Service Biobank, but restrictions apply to the availability of these data, which were used under licence for the current study, and therefore are not publicly available. Data are, however, available from the authors upon reasonable request and with permission of the Finnish Red Cross Blood Service Biobank and FinnGen.
