# Supplementary material for: Blood donor biobank as a resource in personalised biomedical genetic research
Source: Eur J Hum Genet. 2024 Jan 12;34(7):923–31. doi: 10.1038/s41431-023-01528-0 (PMC13342636; doi:10.1038/s41431-023-01528-0)
Supplement: Supplementary file 4 — Supplementary Figure 2 [file 41431_2023_1528_MOESM4_ESM.pdf]

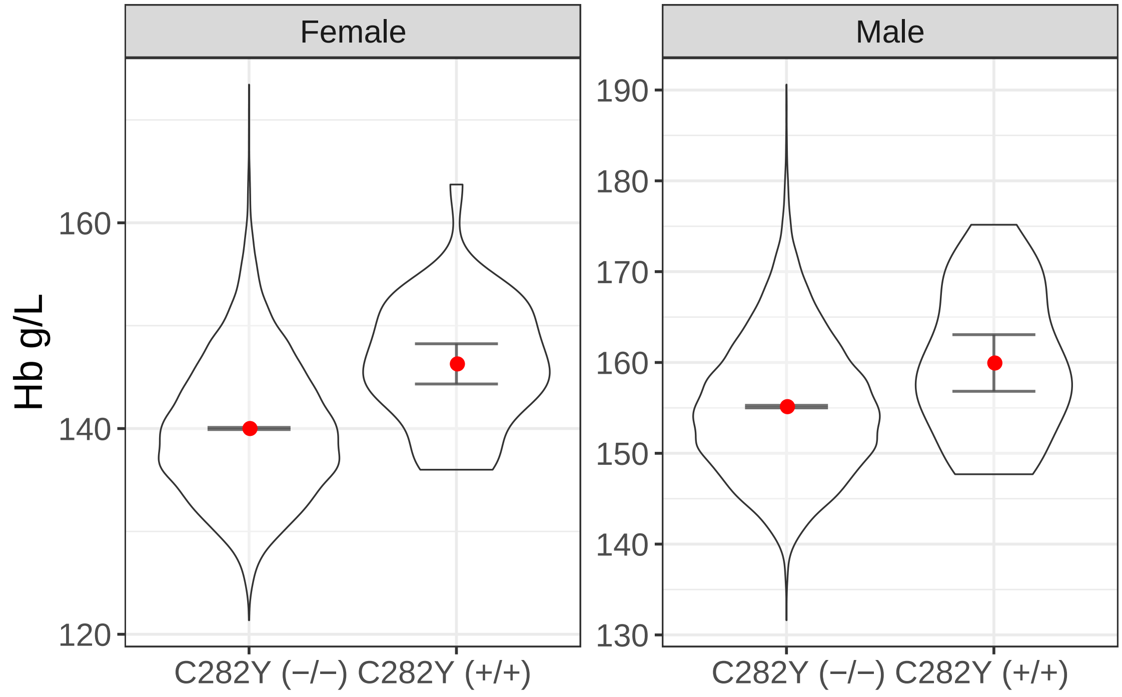

Residuals:				
Min	1Q	Median	3Q	Max
-24.484	-4.996	-0.557	4.457	36.214
Coefficients:	Estimate	Std. Error	t value	Pr(>|t|)
(Intercept)	1.407e+02	7.573e-02	1857.750	< 2e-16 ***
Gender Male	1.503e+01	8.825e-02	170.268	< 2e-16 ***
HFE C282Y (+/+)	5.555e+00	8.667e-01	6.409	1.48e-10 ***
Date of Birth	-1.823e-04	9.513e-06	-19.165	< 2e-16 ***
Donation Count Total	-1.067e-02	1.537e-03	-6.941	3.98e-12 ***
Signif. codes: 0 '***' 0.001 '**' 0.01 '*' 0.05 '.' 0.1 ' ' 1				
p-values are unadjusted				
