# Supplementary figures and images for: Blood donor biobank as a resource in personalised biomedical genetic research
Source: Eur J Hum Genet. 2024 Jan 12;34(7):923–31. doi: 10.1038/s41431-023-01528-0 (PMC13342636; doi:10.1038/s41431-023-01528-0)

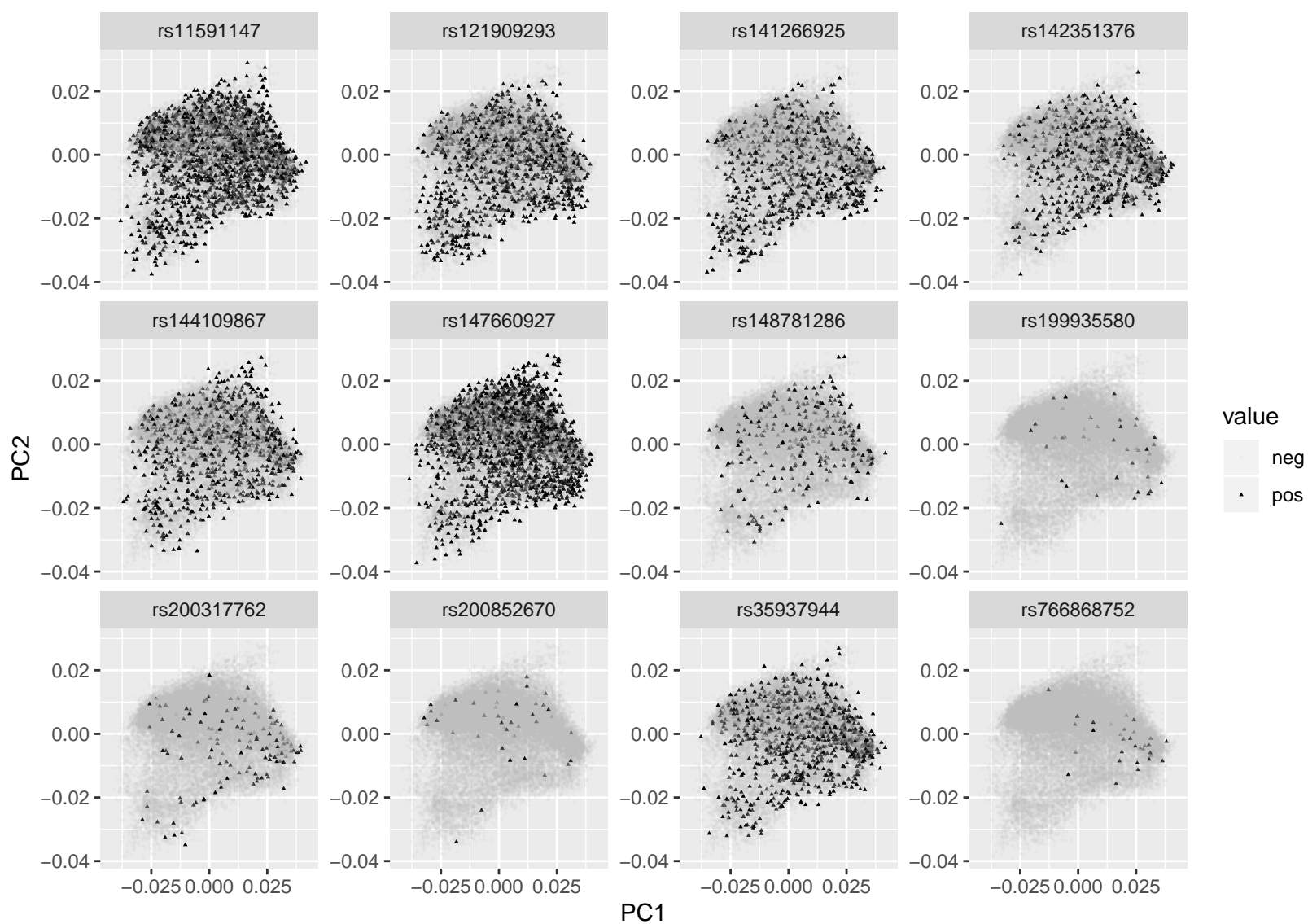

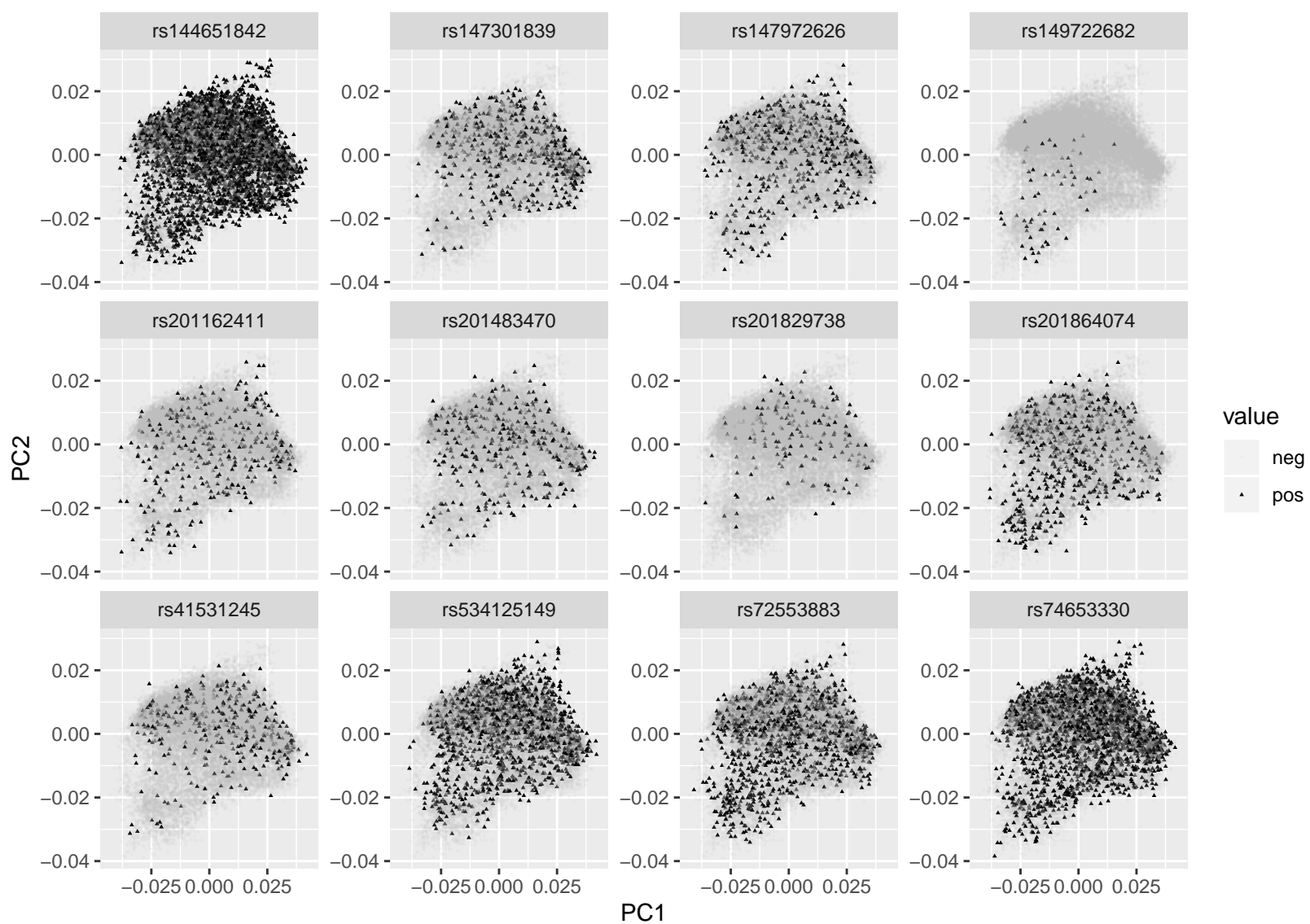

PC2

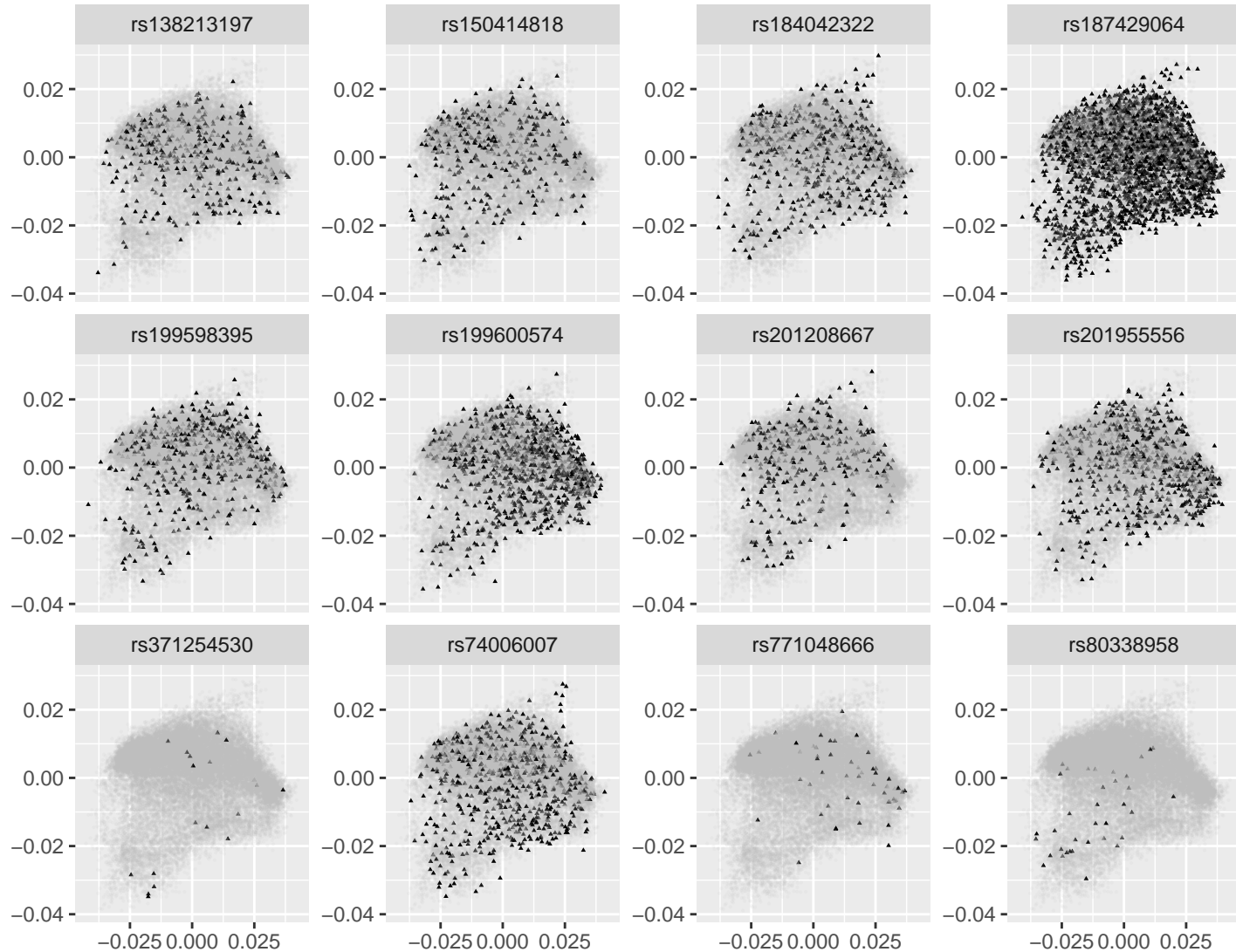

PC1

PC2

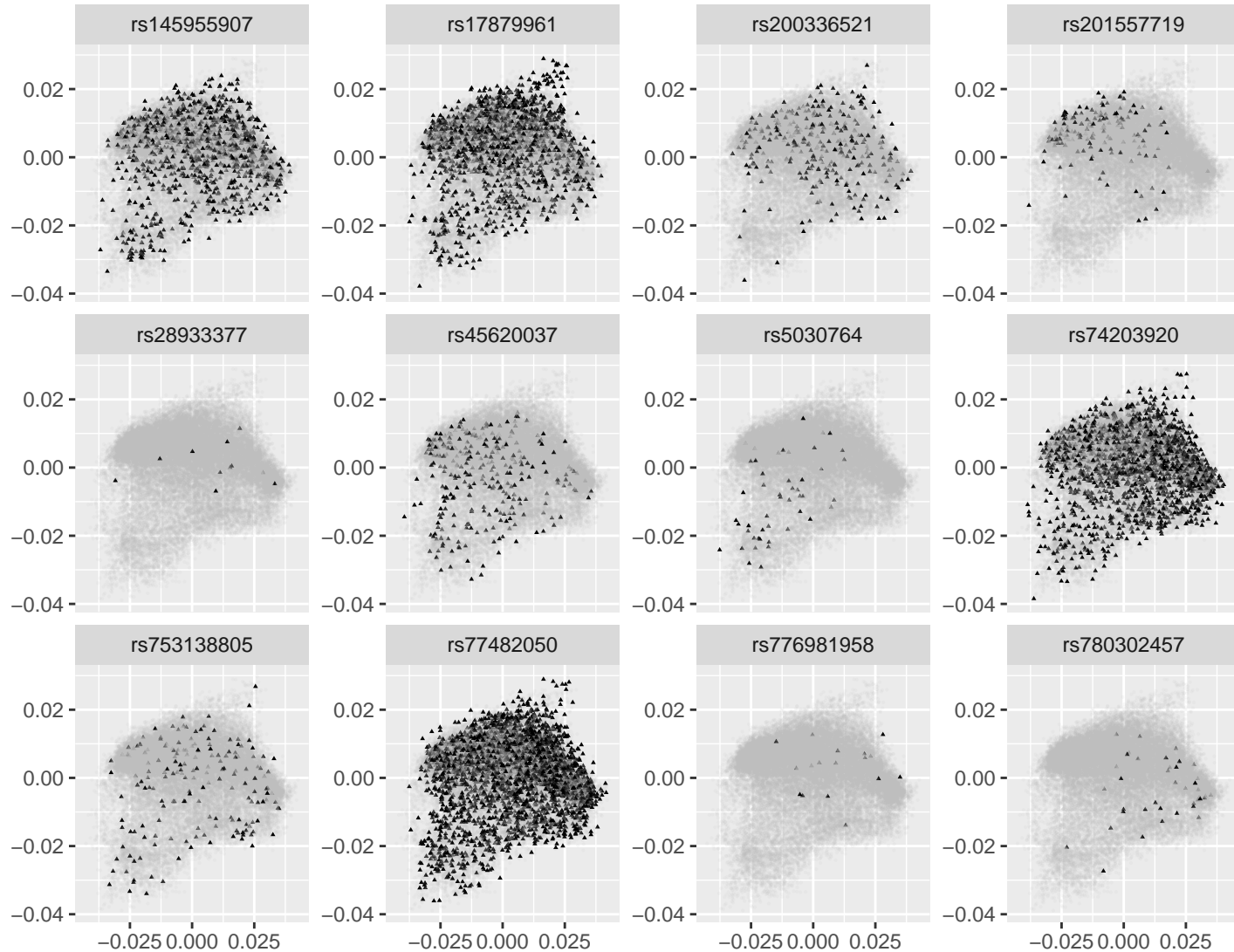

PC2

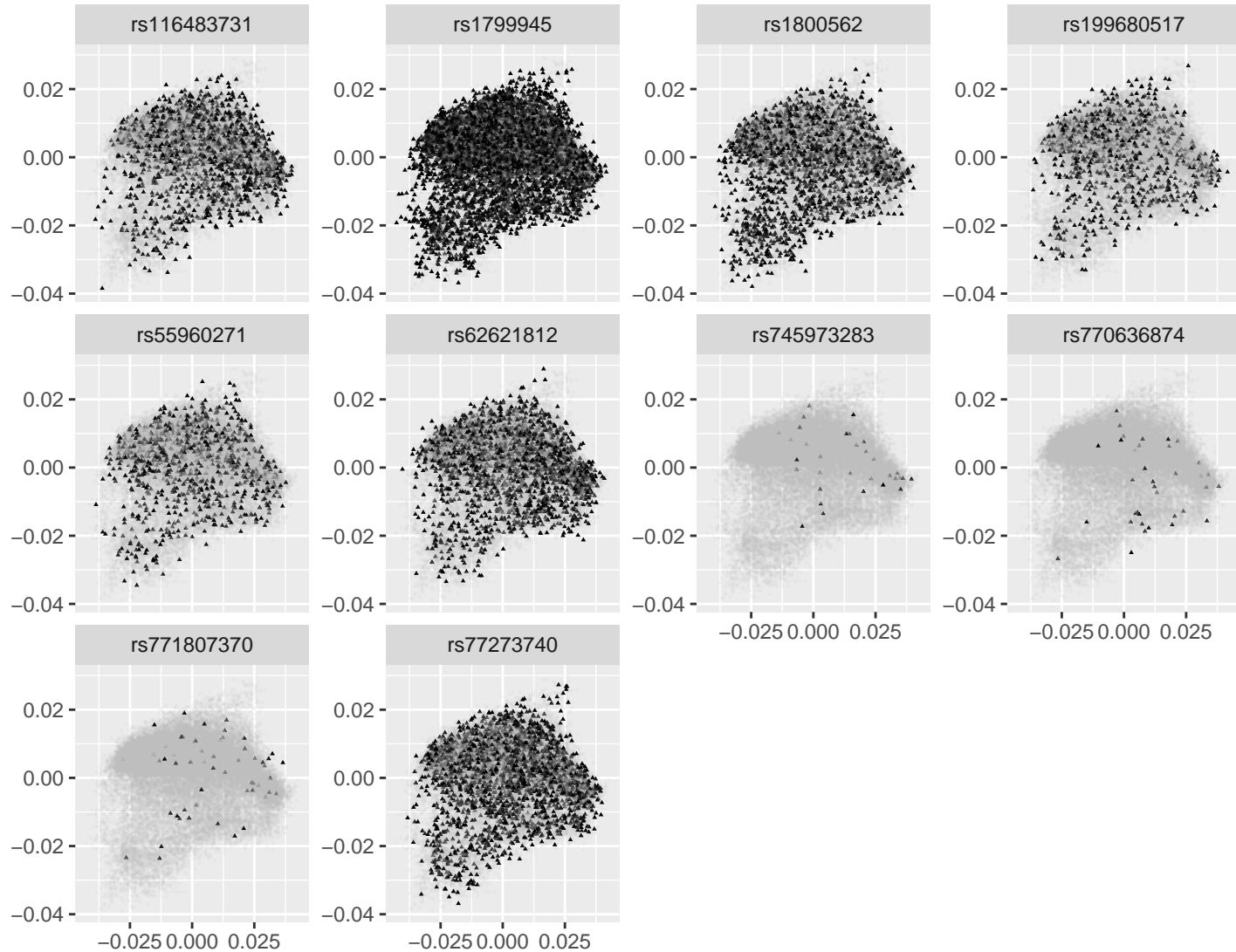

PC1

Supplement: Supplementary file 5 — Supplementary Figure 3 [file 41431_2023_1528_MOESM5_ESM.pdf]
