# Supplementary material for: Blood donor biobank as a resource in personalised biomedical genetic research
Source: Eur J Hum Genet. 2024 Jan 12;34(7):923–31. doi: 10.1038/s41431-023-01528-0 (PMC13342636; doi:10.1038/s41431-023-01528-0)
Supplement: Supplementary file 10 — Supplementary Table and figure legend [file 41431_2023_1528_MOESM10_ESM.docx]

Supplementary Table 1a A list of the 53 rare variants and blood donation associated mutations. The minor allele frequencies in the Blood Service Biobank, the result of Hardy-Weinberg Equilibrium and association found in FinnGen for the rare disease associated variants are listed.

Supplementary Table 1b A list of the Finnish regions and their names in English when applicable.

Supplementary Table 1c A list of frequencies of HFE C282Y, GP1BA Leu129Pro, GP9 Asn45Ser and GP9 Leu40Pro mutations and HLA-DQ2/DQ8 genotype frequencies in each region.

Supplementary Figure 2 Overall mean haemoglobin levels in female and male donors carrying none vs. two copies of HFE C282Y mutation. Median of haemoglobin values in each group and 95 CI is shown in the figure and the results of the linear regression model is shown below.

Supplementary Figure 3 Genome level PCA of each variant in Blood Service Biobank. Neg=donor doesn’t carry any copy of the variant. Pos=donor is homo- or heterozygous for the minor allele of the variant.

Supplementary 4a Mean PC1 and PC2 values shown on a map of Finland based on donor’s home region and PC value.

Supplementary 4b Results of PC1 comparison between donors positive (carrying at least one copy of the variant) or negative (not carrying any copy of the variant) for a specific variant.

Supplementary 4c Results of PC2 comparison between donors positive (carrying at least one copy of the variant) or negative (not carrying any copy of the variant) for a specific variant.

Supplementary Table 5: A full list of FinnGen consortium participants
